# The Encounter of Two Worlds: Divided Narratives of Decision‐Making on Cancer Treatment Between Physicians and Patients

**DOI:** 10.1111/hex.70029

**Published:** 2024-10-02

**Authors:** Weiwei Lu, Dennis Sing Wing Wong

**Affiliations:** ^1^ Department of Social and Behavioural Sciences City University of Hong Kong Hong Kong China

**Keywords:** cancer treatment, goals and obstacles, narrative, person‐centred approach, physician and patient, shared decision‐making

## Abstract

**Introduction:**

Divided narratives pose long‐standing difficulties in physician and patient communication. In decision‐making on cancer treatment, divided narratives between physicians and patients hinder mutual understanding and agreement over the illness and its treatment. For effective decision‐making on treatments, it is necessary to investigate the similarities and differences in these divided narratives.

**Methods:**

This study adopted a qualitative research design of narrative inquiry to examine the data, which included interviews with 32 cancer patients and 16 paired physicians in two hospitals in China. Data analysis was conducted using grounded theory to generate findings.

**Results:**

Both physicians and patients were concerned about goals and obstacles to their decision‐making on cancer treatment. Four common aspects of goal setting were identified from the divided narratives: decision pools, treatment goals, identity practice and preferred identity. Four common obstacles were identified: pains and trust, communication gap, financial issues and complex family. However, the meanings attached to these eight aspects differed between physicians and patients.

**Conclusion:**

Cancer treatment decision‐making is an encounter of the scientific world and lifeworld. A divided narrative approach can identify the similarities and differences in the decision‐making on cancer treatment between physicians and patients. Physicians generally adopt a rational decision‐making approach, whereas patients generally adopt a relational decision‐making approach. Despite the common concerns in their goals and obstacles, physicians and patients differed in their contextualized interpretations, which demonstrates the physicians' and the patients' pursuit of preferred identities in decision‐making. The results of this study provide a new perspective to treatment decision‐making, emphasizing the importance of narrative integration in reaching mutual agreement.

**Patient and Public Contribution:**

The findings were shared with 15 cancer patients and caregivers for feedback and advice in June 2024. This study was also presented at the international conferences of COMET (International and Interdisciplinary Conference on Communication, Medicine, and Ethics) and ICCH (International Conference on Communication in Healthcare) 2023 for continuous feedback and comments.

## 1 Introduction

Different understandings of illness and treatment form divided narratives between physicians and patients in treatment decision‐making. The term ‘divided narratives’ was rarely used before. This study defines divided narratives as the divergent meaning‐making of illness and treatment between physicians and patients. Elliot Mishler proposed that there were two voices in physician and patient communication [1]. The voice of medicine means that physicians create meanings of illness and treatment from medical events in the scientific world. In contrast, the voice of lifeworld means that patients derive meanings from everyday life events. The divided narratives between physicians and patients bring difficulties in mutual understanding and simultaneously produce a power imbalance in communication. The scientific knowledge of medicine defines the sick and the well, leaving the divide between them wide and unbridgeable [2].

Thus, understanding divided narratives is very important because it can demonstrate the complexity of treatment decision‐making through life and medical events [3], reduce the gap between physicians and patients [4] and inspire a pathway to reach power balance and mutual agreement in communication. Particularly in cancer treatment decision‐making, both physicians' and patients' interpretations of illness and treatment were diverse and dynamic due to the profound changes in the scientific world and lifeworld [5, 6, 7, 8, 9].

In the scientific world, the rapid development of evidence‐based medicine [10] and alternative medicine [11] provides more cancer treatment options. In the lifeworld, the global prevalence of the internet and social media produces exploding but poor‐quality information, enriching and complicating patients' understanding of cancer and treatment [12]. Moreover, using technology in both worlds strengthens the divide between physicians and patients. While deep learning technology was used in clinical care to improve standardized cancer screening [13, 14], cancer patients expect technology‐based interventions to address personalized social support and mental well‐being [15, 16].

Divergent and imbalanced ‘voices’ (meaning‐making of illness and treatment) constantly emerge in the evolving medical and life situations, increasing the misunderstanding, mistrust and conflicts on cancer treatment [17, 18, 19, 20, 21] and undermining the effectiveness of treatment.

Abundant research has investigated physicians' and patients' perceptions of cancer and treatment [22, 23, 24, 25] and promoted health information exchange and shared decision‐making to shrink the gap [26, 27]. However, little investigation compares the meaning‐making of illness and treatment between physicians and patients, taking divided narratives as a phenomenon and method in physician and patient communication.

This study explores the divided narratives between physicians and patients to identify the similarities and differences in their meaning‐making of illness and treatment: (1) What are the common elements of decision‐making on cancer treatment between physicians and patients? (2) What are the divergent meanings created by physicians and patients regarding cancer treatment?

## 2 Methods

### 2.1 Research Design, Setting and Sampling

This study is a part of the first author (W. L.)'s thesis project on exploring a narrative‐based model of shared decision‐making on cancer treatment. The first author (W. L.) conducted 3 months of fieldwork to collect data through observation and pair interviews. The fieldwork was in two top‐level hospitals in northwestern China: a state‐owned hospital with over 4000 beds and a private hospital with 3000 beds.

Thirty‐two cancer patients were selected, with their 16 physicians as paired samples. The participants were from four medical departments: gynaecology, medical oncology, surgical oncology and radiation therapy. The inclusion criteria were as follows: (1) the physician and the cancer patient are matched, (2) the patient knows their cancer diagnosis and (3) the physician and the patient are willing and able to participate in the research. The exclusion criteria were patients younger than 18 years old, patients' conditions were not conducive for conversations, patients who were unaware of their cancer diagnosis or physicians who were unwilling or unable to participate.

### 2.2 Data Collection and Procedure

The first author (W. L.) conducted two phone interviews with a physician to understand the cancer treatment decision‐making procedures. Usually, when a patient is diagnosed with cancer, the responsible physician will inform the patient and their family, explain the disease and treatment to them and provide treatment options for the patient.

The interview guide covers five dimensions: (1) Procedures and experiences of decision‐making on cancer treatments, (2) Health information exchange between physicians and patients, (3) Interaction and communication (other than health information exchange) between physicians and patients, (4) Facilitators of and barriers to decision‐making on cancer treatments and (5) Evaluation of treatment decisions. This study's ethics approval was provided by the College Human Subjects Ethics Sub‐Committee from the College of Liberal Arts and Social Sciences, City University of Hong Kong (2021‐22‐CIR3‐6).

The researcher explained the purpose and sampling criteria to all the directors in the medical departments and selected participants on the basis of directors' evaluation. As such, the study can ensure patient safety by considering their well‐being in selection. All participants were well informed of this study's procedures, benefits and risks and provided informed consent.

One‐to‐one in‐person interviews were conducted from May to July 2022. Interviews were arranged in a quiet and designated room in each clinical department. The interviews lasted 30–90 min. Being fully aware of the power imbalance in interviews and regarding interviews as a process of meaning co‐creation [1, 28], the researcher conducted unstructured interviews by following the flow of the conversation. The researcher held a curious and nonjudgemental position to understand participants' language, check their emotional readiness and create safe spaces for them to share their personal stories and opinions. Interview data were deidentified and transcribed verbatim for analysis.

### 2.3 Data Analysis

The first author (W. L.) analysed interview transcripts with NVivo 12. Informed by the grounded theory [28], the first round of open coding [29] was about the concerns in physicians' and patients' treatment decision‐making. The second round of coding identified possible categories of concerns. The ultimate coding schemes were evaluated and synthesized by comparing the similarities and differences between physicians' and patients' narratives. The coding schemes were first generated by interviews with eight pairs of physicians and patients. The ultimate coding schemes were applied to the other 24 interviews, reaching saturation.

The two authors (W. L. and D. S. W. W.) discussed the coding schemes and reached a consensus. To further ensure the credibility of the findings, the first author (W. L.) went back to the field and organised two in‐person group discussions to share the final coding schemes and findings with 11 physician participants for member checking [30]. Physicians resonated with the findings and acknowledged their usefulness.

## 3 Results

The sample comprised 21 female patients and 11 male patients, ages 32–78 years, with the average age being 56.7 years. Patients' occupations included farmers, retired workers, housewives, workers and company staff. In terms of physicians, the study covered five female physicians and 11 male physicians, ages 25–55 years, with the average age being 37.3 years. The average working years of physicians was 12.8 years. A total of 12 types of cancer were covered in this study.

**Figure 1:**
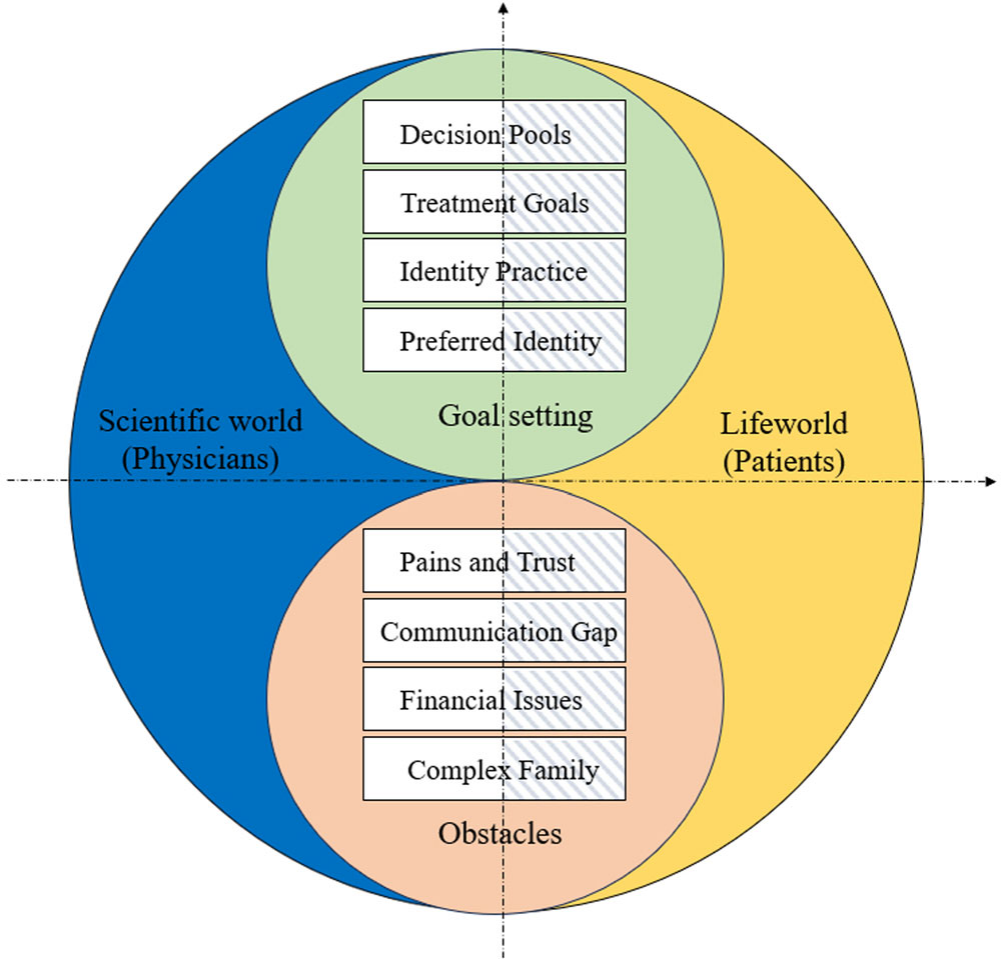
Similarities and differences of divided narratives.

The study identified two common elements in physicians' and patients' divided narratives: Goal setting and the perceived obstacles. Under each element, four subthemes represented identical concerns during cancer treatment decision‐making (see Figure 1). Regarding goal setting, physicians and patients were concerned about the same four issues: (1) Decision pools for goal setting; (2) Treatment goals; (3) Identity practice; and (4) Preferred identity. Regarding the perceived obstacles, physicians and patients recognized four common issues: (1) Pains and trust; (2) Communication gap; (3) Financial issues; and (4) Complex family. However, physicians and patients held divergent interpretations and practices of the above eight common concerns in the divided narratives of cancer treatment decision‐making.

### 3.1 Goal Setting

#### 3.1.1 Decision Pools for Goal Setting

Decision pools refer to the potential cancer treatment choices that physicians and patients have in their scientific world and lifeworld. Although physicians and patients mentioned diverse treatment options, they had different decision pools that included various types and ranges of treatments.

##### 3.1.1.1 Physicians: Plan A or Plan B?

The curability of cancer was a subject of debate among physicians. A group of physicians passively considered cancer treatment a tool to extend life expectancy. Some physicians positively claimed that cancer is curable based on the 5‐year survival rate after treatment. Despite the differing beliefs about cancer curability, all physicians agreed that it was their responsibility to treat patients.

As clinical physicians, we will not give up treatment if we have a glimpse of hope. To cure patients is the original and basic responsibility of a physician.

(Physician 3)

Physicians would prioritize the previous cancer treatment plans for the patients transferred from other hospitals and prepare several treatment options for newly diagnosed cancer patients. Additionally, they aimed for pain management of side effects and disease complications for late‐stage cancer patients.

Physicians believed that cancer treatment decision‐making was a trade‐off among treatment options based on their pros and cons. Therefore, they focused on offering options for patients.

##### 3.1.1.2 Patients: Treatment or Not?

Whether to undergo treatment was an issue that bothered patients, regardless of cancer stages and the type of treatment that they were undertaking or would potentially undertake.

Patients commonly regarded cancer as incurable, and undergoing cancer treatment meant a waste of money, a burden to the family and even dying faster.

Chemotherapy is like a fight with the enemy. When you kill 1000 cancer cells, you will also lose 800 healthy cells. So, when this session is over, I will quit. That is my decision.

(Patient 10)

Other than receiving medical treatment at the hospital, patients also considered alternative treatment options, mainly including Chinese medicine. Patients also considered not receiving any treatment as a treatment option.

For patients, the first choice that they needed to make was ‘to undergo treatment or not.’ Next was the choices among treatment options because patients considered a broader pool of treatment than hospital physicians did.

#### 3.1.2 Treatment Goals

Treatment goals mean that physicians and patients set different goals for cancer treatment. Although physicians aim to achieve medical goals, patients set goals on the basis of relational considerations.

##### 3.1.2.1 Physicians: Medical Goals of Treatment

Physicians set medical goals of treatment to relieve symptoms, control disease progression, reduce side effects and ensure medical safety. The aim was to improve patients' quality of life and ensure that they could die with dignity. Meanwhile, focusing on medical goals relieved physicians' struggle between sympathy and rationality.

Junior physicians (less than 5 years of working experience) constrained their attention to medical goals to avoid coping with the frustration of witnessing so many deaths. Senior physicians adopted biological, religious or philosophical perspectives to deal with patients' deaths. Quick adjustments to patients' deaths helped physicians maintain professional conduct.

After 30 years of working in radiotherapy, I now understand death as a natural life outcome. You can do nothing about it. Nonetheless, I still need 1 or 2 days to digest a patient's unexpected death caused by disease progression.

(Physician 6)

##### 3.1.2.2 Patients: Relational Goals of Treatment

Relational goals refer to the goals that patients want to achieve in relationships. Chinese people emphasize a family‐centred culture. Patients pursued relational goals of treatment over their medical goals. With effective treatment, patients hoped to perform their relational responsibilities.

Although mothers underwent treatment to maintain a body condition to help young generations earn money, fathers stopped treatment to save money for their children's future education or marriage. An exceptional case of a father was a lung cancer patient (Patient 4) who hoped to attain more time through treatment to fix the broken relationship with his son.

Grandparents also contributed to their families in various ways. A pancreatic cancer patient (Patient 30) combined Chinese medicine and chemotherapy to stabilize his condition so that he could solidate his family as a spiritual symbol. A breast cancer patient (Patient 20) repeatedly urged her physician to perform her surgery because she wanted to pick up her granddaughter from kindergarten as soon as possible.

Daughters and sons also directed their treatment goals to relational dimensions. A cervical cancer patient (Patient 14) received the treatment to meet her parents' expectations. A 45‐year‐old man with lung cancer (Patient 28) spent all his money on treatment because he wanted to care for his 80‐year‐old mother.

#### 3.1.3 Identity Practice

Identity practice means how cancer treatment decision‐making orients to the different aspects of identities. Physicians relied on constructing patient identities for ill people to achieve medical goals, whereas patients considered treatment as a process to perform their identities as ordinary people.

##### 3.1.3.1 Physicians: Treatment As Identity Construction

During treatment decision‐making, physicians consciously or unconsciously constructed the patient identity for the people who were newly diagnosed with cancer. This is also called patient socialization, making ill people play a sick role [31].

Some physicians believed that patients would cooperate better when they accepted the sick role.

We will share the diagnosis with patients. If they know the truth, they will be quicker to accept their role as patients and follow our treatment plan.

(Physician 6)

Some physicians considered patients too fragile to know the diagnosis and chose to hide information from patients.

It may not be a good idea to disclose too much to patients. Some patients do not want to be a burden to their families. So, if you tell them the truth, they may cease treatment.

(Physician 16)

The information provision reflected physicians' adaptive strategies to gain patients' adherence by assuming and constructing their patient identity.

##### 3.1.3.2 Patients: Treatment As Identity Performance

Cancer breaks patients' lives but cannot diminish their social identities. The hospital was a place where patients continued performing their identities as ordinary people with different social roles.

I live in the moment; every day is a bonus. I could eat and exercise. I did not expect an affluent life, just a peaceful and healthy life, like any other ordinary person.

(Patient 3)

In the hospital, I can only care for my mother through phone calls, comforting her every 2 days. I am so far away from her, but I cannot leave her alone.

(Patient 28)

Both physicians and patients wanted to fight the cancer. However, their strategies differed. The divergent viewpoints were created through the differing identities of a person as a patient or an ordinary person with an illness.

#### 3.1.4 Preferred Identity

Preferred identities describe the roles that physicians and patients prefer to play in achieving goals. Physicians prefer themselves to be good physicians by improving conformity with professional norms. However, patients preferred to realize other social roles through treatment.

##### 3.1.4.1 Physicians: A Good Physician

Physicians practiced being good physicians. The standards of a good physician encompassed the ability to provide effective treatment for complicated diseases, empathy to feel patients' pains, patience to communicate with patients and compassion to understand and comfort patients' distress.

Matching with the different medical specialties of each oncology department, physicians followed their preferred values of conduct as good physicians.

To cure sometimes, to relieve often, to comfort always. In my 20 years of medical work, I dedicated significant time to communicating with patients and comforting them as I know complete cure cases are rare.

(Physician 4)

Compared with the surgical oncology department, which had more first‐stage cancer patients and emphasized rationality and efficiency, the medical oncology department included more humane care in its practices for late‐stage cancer patients.

##### 3.1.4.2 Patients: A Good Patient?

Although patients were willing to cooperate with physicians to achieve medical goals, they did not claim that they would like to be good patients. Rather, as repeated in the interviews, their preferred identities were mainly associated with their social and familial roles.

They couldn't tell I was a patient if I had make‐up on. I am just a normal person when I am with my friends.

(Patient 10)

My mother is old with anxiety syndrome. I am her only son. I am her significant supporter.

(Patient 28)

I was determined to live a quality life once I knew I had this disease. I don't dare to say that I will live until I am 100 years old. At least, I wish to live independently without bothering my children.

(Patient 2)

### 3.2 The Perceived Obstacles

#### 3.2.1 Pains and Trust

Pains are obstacles for both physicians and patients in achieving treatment goals. Pain management influences the trust between physicians and patients depending on the controllability of pain.

##### 3.2.1.1 Physicians: Pains, Trust and Adherence

Pain management was found to be a double‐edged sword in cancer treatment. If pains were controllable, effective treatment would increase patients' trust in physicians and their adherence. As a medical oncologist, Physician 3 said, ‘The first thing is to reduce the patient's pain. If the pains are controlled well, patients will have high adherence to the treatment.’ If the pains were uncontrollable, patients may choose the conservative treatment (simply doing nothing to treat the cancer) over continuing with the medical treatment.

Pains included the suffering from cancer and the pains of the treatment itself. Surgery, chemotherapy and radiation therapy all caused pain. A radiation oncologist, Physician 10, felt stressed about a patient who was in too much pain to sustain the treatment. The same as other physicians' sharing about troublesome patients, the pain of the treatment was taken for granted and deemed less important or unnecessary to manage/address compared to cancer pains.

##### 3.2.1.2 Patients: Dual Pains

The unbearable pains from chemotherapy, surgery and radiation therapy made patients hesitant to continue treatment. Patients suffered from dual pains: physical and mental pains.

There is 30% physical pain and 70% mental distress. I have a colleague who was diagnosed with pancreatic cancer. He immediately collapsed after the diagnosis. Within a month, he passed away from fear.

(Patient 22)

Patients became sad, anxious, depressed, resistant or more irritable when facing their existential crisis.

I felt sad to mention this (cancer). It is the time for me to enjoy life. My children and my wife felt sad for me too. After the diagnosis, I was too depressed to eat or sleep.

(Patient 29)

Patients felt ashamed when people around them regarded cancer as a ‘bad’ disease.

I did not tell any of my friends about my disease. How will they look at me? They probably will say she must have done something bad so that she got cancer.

(Patient 6)

#### 3.2.2 Communication Gap

The communication gap demonstrates the different aspects of communication that physicians and patients identified as obstacles. Physicians applied various communication methods to explain the mechanism of disease and treatment, but patients feared talking to medical authorities.

##### 3.2.2.1 Physicians: Making Patients Understood

Facing health literacy barriers and cultural and religious differences in communication, physicians used plain language, metaphors and drawings to explain the treatment options, risks and benefits to patients. Physicians believed that they were responsible for avoiding conflicts and complaints by persistently and dutifully maintaining a good attitude in communication.

If they cannot understand the treatment at the first attempt, we continue to explain until they get it.

(Physician 6)

We could explain the disease to them at different places. You can talk to them in the office; you can talk to them in the ward. They can sit down to listen to me. Possessing a good attitude would improve the effectiveness of communication.

(Physician 14)

##### 3.2.2.2 Patients: Talking to the Authority

However, patients were hesitant to consult directly with physicians. Cancer patients encounter many uncertainties and puzzles produced by the treatment and its risks and side effects. What prevented patients from consulting their physicians about these uncertainties was not the difficult medical terms or poor health literacy but their fear of challenging or disturbing the authority.

Patients respected physicians as busy experts who might consider their questions naïve, meaningless and a waste of time. They believed in the physicians' professional knowledge and counted on their morality. Therefore, they were cautious not to cross a boundary that may ruin the treatment plan and make physicians unhappy.

I must believe in physicians. It is the same for every patient. Physicians are supposed to take care of patients. The most important thing is their morality. I believe all the physicians in hospitals are good. Therefore, we need to obey their instructions. No doubt.

(Patient 23)

#### 3.2.3 Financial Issues

Physicians perceived financial affordability as a determinant of treatment decisions, whereas patients evaluated treatment on the basis of its worthiness. Whether to accept a treatment depended on the value of this choice that represented the patient's priorities, preferences or bias in social beliefs and norms. The paired cases discussed below most appropriately demonstrated the fundamental roots of the divergent interpretations of financial issues.

##### 3.2.3.1 Pair Cases: Whether It Is Affordable or Valuable?

Physician 6, a radiation oncologist, believed that patient 10 stopped treatment because of money when she learned that the patient was a single mother and did not earn much. Conversely, the patient shared that she was afraid of the side effects of chemotherapy, which she believed would shorten her life.

In the radiation department, the husband of patient 14 complained about a nurse's bad attitude and, thus, wanted to be discharged. The physician in charge judged the problem to be that the husband could not afford more expenses on his wife's treatment. However, after the husband shared how he had taken his wife to various hospitals over the past 8 years and spent around CNY 400,000 (USD 55,000), physician 9 realized that he misunderstood the husband (In this hospital, some cancer patients could not afford around USD 2700 to take cancer treatment). The implicit need from his complaints might be more comfort from the medical service.

Physician 16 thought financial burden was a key factor in treatment decision‐making. Nevertheless, his patient's consideration went beyond money.

As a farmer, I do not have the money even to afford a bottle of water. But with this disease, I need plenty of money for treatment. My father got cancer last year. I spent a lot of money on him, which financially burdened me. This year, I got sick. I have an old mother to care for and two children to nurture. If I don't get treated and I die, my family will collapse.

(Patient 28)

#### 3.2.4 Complex Family

Complex family covers various family situations that make cancer treatment decision‐making mingled with risks, conflicts and meanings. Complex families usually represented higher medical risks in physicians' opinions, but patients still needed their family members' involvement to construct the meaning of treatment.

##### 3.2.4.1 Physicians: Risk Control of Treatment

Complex families brought risks and uncertainties in treatment. One problem was the lack of family support. Physicians expected family members to care for cancer patients' psychological problems to support their treatment or mitigate the risk of suicide. Another risk was the involvement of multiple family members. Family members might become impatient if the treatment was less effective than expected. They might transfer their anger or disappointment to physicians, causing medical conflicts. In addition, disagreements among family members made it harder to arrive at a treatment decision.

Families with multiple children are often risky. It is strange that daughters, rather than sons, usually bear the financial expense of medical treatment if their economic situations allow it. If there are multiple sons, the problem gets complicated. Some would like to pay while others would not; there will be conflicts. In some cases, they quit the treatment because of these complex family issues.

(Physician 10)

##### 3.2.4.2 Patients: Family Decision‐Making of Treatment

For patients, family always played an essential role in decision‐making, be it to continue or cease treatment. Patients felt supported, cared for and protected when they saw that family decision‐making made their children more mature, their marriages more solid and their relationships more precious. The family was a firewall for patients to escape the fear of death.

However, when family members quarrelled with each other about the fortune allocation of the dying patient, had disputes over the treatment expenses or leveraged the disease to negotiate the power status in the family, patients suffered more rather than benefiting from family decision‐making.

It was meaningless for me to live long because of the conflicts with my husband. I did not want to live. But my relatives comforted me. They called me every day to encourage me. So, I decided to live for my children and myself.

(Patient 21)

## 4 Discussion

### 4.1 The Encounter of Two Worlds

The findings demonstrate that decision‐making on cancer treatment between physicians and patients is an encounter of the physician's scientific world and the patient's lifeworld.

The different knowledge bases from the two worlds produce corresponding cognitions, emotions and decision‐making styles. Physicians apply their medical knowledge to the treatment plans to achieve goals that make medical sense. Though they struggle with sympathy and emotional burdens, physicians set up a professional boundary of rationality [32, 33]. Physicians' treatment decisions strictly follow cancer treatment guidelines. Therefore, physicians are found to mainly adopt the rational decision‐making approach.

Patients play the sick role and are socially acknowledged as weak, fragile, helpless and emotional [34, 35]. Because of the illness stigma and sadness that they have witnessed in their lifeworld, patients may first ascribe a negative or even traumatic meaning to their illness and treatment and feel passive towards their diagnosis [36]. However, with the support from familial, cultural or social relationships [37, 38, 39], they gradually develop the base of treatment decisions by creating positive meanings of illness and treatment. Therefore, patients are found to mainly adopt a relational decision‐making approach.

The different knowledge bases between two parties produce a power imbalance in communication [2]. Scientific knowledge of cancer and treatment is socially accepted as standard, legitimate and accountable, whereas life knowledge is individual, diverse, improvisational and thus unaccountable. Physicians' voices dominate the form and content of medical conversations, being inattentive to patients' voices regarding the history and course of the cancer symptoms and their effects on their lives [1].

### 4.2 Behavioural and Existential Dimensions in Divided Narratives

Contributing to the existing cognitional and emotional chasms, this study discloses the behavioural and existential differences in divided narratives. The same structure of goal setting and obstacle perceptions in physicians' and patients' narratives orient their different actions towards illness and treatment. The tension between goal setting and obstacles intensifies physicians' and patients' existential concerns about the fundamental problems of uncertain cancer curability, emotional vulnerability, power, trust and meaning of life [40].

Moreover, the study finds that the differences in identity construction navigate all four dimensions of divided narratives. Identity here is a cyclical way in which individuals construct knowledge of the world and themselves [41]. Different knowledge bases direct physicians' and patients' identity construction and predict their different cognitive, emotional, behavioural and existential responses to the illness and its treatment. And in return, these responses continue shaping their identities in the scientific world and the lifeworld.

Through treatment decision‐making, physicians and cancer patients not only play their social roles as physicians and patients but also pursue their preferred identities as good physicians, family members or good persons. Patients are persons in the clinic [42], physicians are also persons in the clinic. Preferred identity construction is a life‐long trajectory of meaning‐making, which develops agency for physicians and patients to engage in treatment decision‐making and take action. These insights connect the divided narrative perspective to the umbrella of person‐centred care [43], which emphasizes the result of care on a more meaningful life than a functional life [44].

### 4.3 Information Exchange and Narrative Integration

This study further develops a narrative perspective of shared decision‐making. Shared decision‐making is a trend of treatment decision‐making that aims to achieve mutual agreement on treatment based on patients' preferences. [45] With the recognition of the information asymmetry between physicians and patients [46, 47], the existing research made efforts to improve physicians' communication competency [48, 49], increase patients' knowledge by education [50, 51, 52] and develop decision aids to facilitate information exchange [53, 54].

However, information exchange neglects the autonomous and divergent interpretations of information between physicians and patients. Also, information increase cannot reduce the power imbalance between physicians and patients [55]. Further, it is the conversation not the information that can reach the narrative integration between physicians and patients [56], acknowledging them as equal co‐authors to co‐construct a new narrative as a treatment decision. Notably, this study found that the shared principle of treatment decision‐making between physicians and cancer patients is the pursuit of preferred identity, which can be the core orientation of shared decision‐making through narrative integration.

## 5 Conclusions

### 5.1 Strengths and Limitations

The strength of this study is the divided narrative approach, which is fully aware of the context in data collection and analysis. Context refers to physicians' scientific world and patients' lifeworld behind their cancer treatment decision‐making.

Current medical research usually investigates physicians' and patients' differing thoughts separately, neglecting the complex meaning‐making process in their unique contexts in the scientific world and lifeworld. As a result, those findings are incomparable and provide limited implications for practice.

The narrative approach is an effective research method to explore stakeholders' experiences and interpretations [3, 57]. A divided narrative approach comprehensively includes the contextual factors related to events, emotions, rationalities and meanings in physicians' and patients' worlds, presenting a more authentic reality. By pair interview, this study controls the research context within the same event, making the perspectives comparable, verifying the divergent interpretations of information and highlighting the importance of investigating divided narratives between physicians and patients.

All the above efforts strengthen the credibility of the data and findings, enriching this study's implications. For research, this study articulates the underlying principles and values of coproduction and codesign research in shared decision‐making on cancer treatment [58]. For medical education, the similarities and differences in divided narratives between physicians and patients can be included in the medical curriculum to better inform physicians and engage patients in treatment communication. For practice, a range of supportive interventions can be developed to amplify patients' voices and improve mutual understanding in treatment decision‐making.

The limitation of the study is its generalizability. A divided narrative approach can be applied to physician and patient communication in various contexts. The findings of this study can significantly contribute to implementing shared decision‐making in Chinese communities. However, because the content of two worlds demonstrates the unique social, cultural and economic systems in a specific medical context, the similarities and differences in divided narratives may vary from context to context.

### 5.2 Concluding remarks

Facing the uncertainties brought about by the ever‐changing events in the scientific world and lifeworld, it is essential to know how physicians and patients interpret the meanings of these events. A divided narrative approach can explore physicians' and patients' meaning‐making of illness and treatment in their complex contexts, providing compelling evidence for us to understand the ‘meaning gap’ between physicians and patients. The narrative perspective indicates an attention shift from ‘information’ to ‘information interpretation,’ which can further respond to the current technology trend in the scientific world and lifeworld.

## Author Contributions

**Weiwei Lu:** conceptualization, methodology, software, data curation, formal analysis, investigation, visualization, project administration, writing–review and editing, writing–original draft, validation, resources. **Dennis Sing Wing Wong:** supervision, data curation, writing–review and editing, validation, methodology.

## Ethics Statement

Ethics approval was provided by the College Human Subjects Ethics Sub‐Committee from the College of Liberal Arts and Social Sciences, City University of Hong Kong (2021‐22‐CIR3‐6).

## Conflicts of Interest

The authors declare no conflicts of interest.

## Data Availability

Due to participant privacy, the data are confidential.
